# Biosimilar competition in European markets of TNF-alpha inhibitors: a comparative analysis of pricing, market share and utilization trends

**DOI:** 10.3389/fphar.2023.1151764

**Published:** 2023-04-21

**Authors:** Elif Car, Arnold G. Vulto, Mark Van Houdenhoven, Isabelle Huys, Steven Simoens

**Affiliations:** ^1^ Department of Pharmaceutical and Pharmacological Sciences, Leuven, Belgium; ^2^ Hospital Pharmacy, Erasmus University Medical Centre, Rotterdam, Netherlands; ^3^ Sint-Maartenskliniek, Nijmegen, Netherlands

**Keywords:** biosimilar, market dynamics, pricing, utilization, competition, infliximab, etanercept, adalimumab

## Abstract

**Background:** Factors like the number of biosimilar competitors and competitive pricing strategies from originator companies may influence price competition and biosimilar uptake.

**Objective:** The aim of this study was to analyze multiple facets of biosimilar competition of TNF-alpha inhibitors in Europe by exploring the existence of a biosimilar first-mover advantage, pricing strategies of originator companies, and the evolution in patient access.

**Methods:** Sales and volume data on biosimilar and originator infliximab, etanercept, and adalimumab between 2008 and 2020 were provided by IQVIA. Countries included 24 European Union Member States, Norway, Switzerland, United Kingdom, Serbia, and Bosnia and Herzegovina. Sales value was expressed as ex-manufacturer price per defined daily dose (DDD), and volume data were transformed into the number of DDDs per 1,000 inhabitants per day. Descriptive analyses were conducted based on the evolution in price per DDD, trends in biosimilar and originator market shares and utilization trends.

**Results:** Market entry of the first biosimilars of infliximab and adalimumab resulted in a decrease of the volume-weighted average price (VWAP) per DDD by 13.6% and 0.9% on average, whilst the second biosimilars resulted in a decrease by 26.4% and 27.3%, respectively. The first and second etanercept biosimilars generated a similar decrease in the VWAP per DDD by 9.3% and 9.1% on average, respectively. Average market share captured by the first biosimilars was at least twice as large as the second biosimilars for all molecules. In addition, sharp reductions in price per DDD of Humira^®^ in most countries indicated a pricing strategy resulting in low uptake of adalimumab biosimilars. Lastly, utilization of infliximab, etanercept, and adalimumab following biosimilar entry increased by an average of 88.9%, 14.6%, and 22.4%, respectively. However, introduction of (multiple) biosimilar competitors did not necessarily translate into increase in treatment access for all three molecules across some European countries indicating a shift in utilization from one molecule towards the other(s).

**Conclusion:** Overall, this study revealed that biosimilar entry results in increased utilization and price reduction, although at a heterogenous rate among TNF-alpha inhibitors. Observed trends in market shares indicate a biosimilar first-mover advantage whereas pricing strategies considered to be anti-competitive can limit market uptake.

## 1 Introduction

Biological medicines, or biologics, have had a tremendous positive impact on patients suffering from cancer and various autoimmune disease areas like rheumatology, gastroenterology, dermatology, and endocrinology (Putrik et al., 2014; Verrill et al., 2019). Driven by their increasing costs and substantial increase in utilization, biologics accounted for 34% of medical expenditure corresponding to €8.8 billion spend in Europe in 2021 (IQVIA, 2021). Given the high development costs of these medicines, partly due to the complexity of the manufacturing process, third-party payers have been facing difficulties to manage the growing budget impact threatening patient access and sustainability of their healthcare systems. Especially in Central and Eastern European countries (CEE), biologics are usually considered not cost-effective (Inotai et al., 2018). The loss of exclusivity for an already approved biological medicine (i.e., originator or reference product), has made it possible to introduce less costly alternatives, biosimilars, to the market. Unlike generic medicines designed and developed to be exact copies of small chemical molecules, biosimilars are highly similar but not identical versions to the originator product. According to the European Medicines Agency (EMA), a biosimilar medicine is defined as “*a biological medicinal product that contains a version of the active substance of an already authorized original biological medicinal product (reference medicinal product) in the European Economic Area. Similarity to the reference medicinal product in terms of quality characteristics, biological activity, safety and efficacy based on a comprehensive comparability exercise needs to be established*” (EMA, 2014).

**FIGURE 1:**
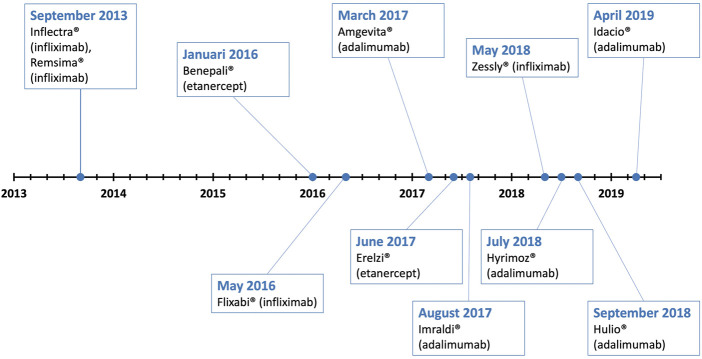
Timeline of dates of biosimilar TNF-alpha inhibitors approval by EMA.

Among biological medicines, tumor necrosis factor (TNF)-alpha inhibitors (infliximab, etanercept, adalimumab, certolizumab pegol, and golimumab) are widely used to treat inflammatory or autoimmune diseases such as rheumatoid arthritis, Crohn’s disease, ulcerative colitis, psoriasis, psoriatic arthritis, ankylosing spondylitis, juvenile idiopathic arthritis, and hidradenitis suppurativa (Jang et al., 2021). These medicines present a continually growing high value market with currently numerous biosimilars approved and are among the best-selling biologics worldwide, including the world’s best-selling medicine (Humira^®^). Global sales of Humira^®^ exceeded $20 billion in 2020 which is approximately one-third of total autoimmune sales worldwide (Troein et al., 2019; Amgen, 2021). Timeline of the marketing authorization dates of all EMA-approved infliximab, etanercept, and adalimumab biosimilars is shown in Figure 1. Following patent expiry of the originator infliximab (Remicade^®^) in 2013, two infliximab biosimilars, Remsima^®^ and Inflectra^®^, were approved by EMA on 10 September 2013. Following approval of the first biosimilars, Flixabi^®^ and Zessly^®^ were introduced to the European biosimilar market in May 2016 and May 2018, respectively. Etanercept biosimilars included in the analysis, Benepali^®^ and Erelzi^®^, were granted marketing authorization in January 2016 and June 2017, respectively. Lastly, patent expiration of the originator adalimumab (Humira^®^) in 16 October 2018 was preceded by authorization of four adalimumab biosimilars spread over several months from each other: Amgevita^®^ (March 2017), Imraldi^®^ (August 2017), Hyrimoz^®^ (July 2018), and Hulio^®^ (September 2018). EMA-approval of Idacio^®^ in April 2019 was followed by Amsparity^®^ in February 2020. Two of adalimumab biosimilars, Amgevita^®^ and Imraldi^®^, were directly available on the market after patent expiry of Humira^®^ on 16 October 2018.

A key benefit of biosimilar competition is generating cost-savings. Biosimilar entry may result in price competition leading to price reductions of the originator biologic as well as the whole product line (originator and its biosimilars). With biosimilars, a brand-to-brand competition can be observed, different from generic markets with small molecules due to the lower price differences with the originator product suggesting weaker price competition. Biosimilar prices are in general 10%–35% lower compared to their originator biologic counterparts. Despite this, discounts up to 80% have been reported suggesting that absolute savings from biosimilars are significantly reducing healthcare spending as highlighted by €18 billions of cumulative savings in Europe since the approval of the first biosimilar (Farfan-Portet et al., 2014; Moorkens et al., 2017; Hordijk, 2019; IQVIA, 2021). Current experience from Europe has demonstrated that subsequent cost savings generated from biosimilar competition in turn broaden and increase access to biologic medicines to treat a larger number of patients. Furthermore, increased access leads to improved health outcomes by initiating treatment in an earlier phase of the disease as well as improved medication adherence resulting from reduced patient co-payments (Albrecht et al., 2015; Medicines for Europe, 2022). Although improving patient access is one of the primary key benefits of biosimilar competition, access restrictions are still reported in Central and Eastern European (CEE) countries indicating lower usage of biopharmaceuticals and inconsistent biosimilar uptake (Inotai et al., 2018; Inotai and Kaló, 2019; IQVIA, 2021).

Although biosimilars have shown major benefits in terms of reducing pressures on healthcare budgets, their commercial future remains uncertain. Current literature indicates discrepancies in penetration rates of biosimilars between, but also, within European countries (Rémuzat et al., 2017a; Moorkens et al., 2019a; Moorkens et al., 2019b; Moorkens et al., 2020; Troein et al., 2020). Furthermore, previous studies highlighted variations in biosimilar uptake related to differences in market dynamics between product lines as well (Moorkens et al., 2019a; Moorkens et al., 2019b; Moorkens et al., 2020; Moorkens et al., 2021). Looking at the evolving competitive landscape of off-patent biologics and biosimilars markets, specific factors, and barriers which highly influence, limit or delay uptake of biosimilars, have been a focus of researchers in recent years. For instance, off-patent biologics and biosimilars’ uptake and utilization might be affected by decisions on pricing and reimbursement on a local level, differences in, but also, the lack of incentive policies which are under the responsibility of the Member States.

With numerous biologics and blockbuster brands facing patent expiry, a window of opportunity opens up for biosimilar manufacturers, with time to market being a key driver of biosimilar uptake (Niazi, 2022). Evidence from the United States on generic pharmaceuticals has shown that time to market and even the order of market entry is associated with market share. It has been reported that the first generic entrant benefits from a 80% higher market share than the second and further entrants during the first 3 years since market entry. (Yu and Gupta, 2014). Another research work reported that the date of market launch (i.e., date of the first identified sale) of the first biosimilar entrant was positively correlated to market uptake indicating that a longer existence on the market presents a greater market share advantage against late entrants (Rémuzat et al., 2017a). A study reported in 2016 found that early biosimilar entrants gained the largest share of the market in comparison to second or late entrants, similarly to what has been observed in the generic medicines market (Chen et al., 2021). It has been suggested therefore that a biosimilar manufacturer as the first entrant can profit from a competitive advantage over late entrants. This is known as the “first-mover advantage” which indicates that the first manufacturer entering the market has the potential to claim the largest market share (Endrenyi et al., 2017). However, the existence of a first-mover advantage of biosimilars and its impact on market share has not been extensively explored yet in Europe.

The response of originator companies to the market entry of biosimilars is another determinant of biosimilar uptake that could potentially jeopardize access to biosimilars. A variety of strategies can be adopted by originator brands to protect their share of the market to other competitors or generic entry which is not unique to biological medicines only. Examples seen in Europe include product modifications by introducing second generation products or reformulations, improvements in dosing, supporting devices, patent thickets, and price competition (Troein et al., 2020; Vidal et al., 2020). However, several tactics to delay biosimilar entry or reduce their market potential have been scrutinized by regulators (CMA, 2019; Hoen, 2019; Autoriteit Consument and Markt, 2022). With the latter, a key area is the use of aggressive pricing strategies from originator patent holders to further limit biosimilars entry with notorious examples that have caught attention in recent years. In 2015, the pharmaceutical company Merck Sharp & Dohme Limited, which sells the originator infliximab (Remicade^®^), was suspected of offering discounts where the level of discounts was related to the volume purchased by the National Health Service in the United Kingdom with the aim to reduce or delay market entry of biosimilars. If successful, benefits of annual cost savings of approximately 100£ million from lower priced biosimilars would have not been realized. In the Netherlands, an investigation by the local competition authority ACM was initiated against the former patent owner of Enbrel^®^ accusing the company of offering exclusive discounts to hospitals if they purchase the originator biologic above a pre-specified percentage (Autoriteit Consument and Markt, 2022). Likewise, discounts of up to 89% were offered by AbbVie to hospitals in the Netherlands on condition that all patients were treated with Humira^®^. As a result, AbbVie was able to retain a large share of the market whereas two of the four adalimumab biosimilar manufacturers combined were able to capture only 15% of the market while the other two provided each for one hospital (Hoen, 2019). A recent US study has estimated the impact of biosimilar competition on prices and utilization of several biological medicines and reported two heterogeneous pricing responses of originator companies following biosimilar entry. In the first scenario, the originator may react to biosimilar entry by reducing its price as multiple biosimilars enter the market to retain a considerable size of the market. In the second scenario where the originator does not engage a great deal in price reduction, reduced prices may be the result of large shifts in volume from the originator to its biosimilars (Frank et al., 2022). To date, within the class of TNF-alpha inhibitors, a correlation between price reductions from the originator biologic and lower uptake of biosimilars has been demonstrated. However, to our knowledge no study has investigated the influence of various pricing strategies of originator companies on biosimilar uptake in Europe (Troein et al., 2017).

The purpose of this study is to analyze multiple facets of biosimilar competition in European markets of TNF-alpha inhibitors by exploring the existence of a biosimilar first-mover advantage, pricing strategies of originator companies, and utilization trends. The added value of this study lies in the comparative analysis in pricing, market share and utilization trends across 29 countries over a 12-year period. This analysis provides insights in the market dynamics of TNF-alpha inhibitors in Europe and may aid decision makers to develop policy measures that support the potential of biosimilar competition.

## 2 Materials and methods

### 2.1 Data

Retail and hospital sales value and volume data on off-patent TNF-alpha inhibitor biologics and biosimilars on a yearly basis from 2008 to 2020 were provided by IQVIA. With a view to investigate biosimilar competition, the analysis included infliximab (L04AB02), etanercept (L04AB01), and adalimumab (L04AB04) The other two TNF-alpha inhibitors (certolizumab pegol and golimumab) were not selected since biosimilars of these molecules were neither available nor approved in Europe during the study period.

Data were obtained for European Union Member States, Norway (member of the Economic European Area), Switzerland, United Kingdom, and Republic of Serbia and Federation of Bosnia and Herzegovina (entities of State of Bosnia and Herzegovina). Data from the Netherlands and Denmark were not available.

Although data were available from 2008 until 2020, the study period ended in 2019 with a view to eliminate any impact from the COVID-19 pandemic on utilization trends.

### 2.2 Variables

Sales value was expressed as total sales (ex-manufactory level) in US dollars at the exchange rates in effect at the time the sales were made. Sales volume included the number of packages (units) sold and the total number of defined daily doses (DDDs) per year (WHO, 2022). To control for differences in population size and to allow international comparison, volume data were transformed into the number of DDDs per 1,000 inhabitants per day. Data on population size were obtained from EuroStat.

The price of the originator biologic in each year was expressed as ex-manufacturer price (without accounting for discounts) per DDD. First the price per day DDD calculated from dividing annual sales by the annual number of DDDs. In addition, an annual volume-weighted average price (VWAP) per DDD was calculated for each product line (i.e., originator and its biosimilars). The VWAP per DDD reflects the average price of a molecule adjusted for its volume.

### 2.3 Descriptive analyses

With respect to the first-mover advantage, the evolution in the VWAP per DDD of a product line since biosimilar entry and the trend in biosimilar market shares were analyzed with a view to explore whether the first biosimilar spurs the largest reduction in the VWAP per DDD (originator and its biosimilars) and retains the largest biosimilar market share, respectively. Since data are presented on a yearly basis, the market entry of the first individual biosimilar could not be differentiated from the next biosimilars in most countries. The latter applies specifically to the first infliximab biosimilar CT-PT13 which was co-marketed by Hospira (Remsima^®^) and Celltrion (Inflectra^®^) and the first adalimumab biosimilars, such as Amgevita^®^ and Imraldi^®^, which entered the market within few months of each other directly after patent expiry of the originator brand (Humira^®^).

The order of entry, meaning each year of biosimilar entry, will be described further in the paper as “an event”. This refers to the year in which one or multiple biosimilars entered the market (which may differ per country). Each event of biosimilar entry was compared to the previous event. For instance, the VWAP per DDD in the year during which the first biosimilar(s) entered the market is compared to the VWAP per DDD in the previous year (i.e., the year before biosimilar entry). To make a comparison between all three molecules, the analysis will be restricted to only the first and second event of biosimilar entry.

Pricing strategies of originator companies were examined by analyzing the evolution in price per DDD of the originator and the VWAP per DDD of each molecule once biosimilars enter the market. Additionally, trends in biosimilar and originator market shares were also investigated. Market share was expressed with reference to the total market (originator and its biosimilars). Lastly, since lifecycles differ among these molecules with different timepoints of loss of exclusivities, the minimum period in which biosimilars entered the market for infliximab, etanercept, and adalimumab was chosen to be 2 years. Therefore, the average change in price per DDD and VWAP per DDD 2 years since biosimilar entry (versus year before biosimilar entry) was also calculated.

Utilization trends were measured by three indicators: a) the change in volume in 2019 versus year before biosimilar entry, b) the average annual change in volume pre-launch, calculated from 2010 until year before biosimilar entry, and c) the average annual change post-launch, calculated from year of biosimilar entry until 2019. The analysis was conducted separately for all three molecules (infliximab, etanercept and adalimumab) in each country. We applied a statistical outlier removal method to flag potential outliers from the data using the interquartile range (IQR). Extreme outliers were defined as observations that fall more than three times the interquartile range below the first quartile or above the third quartile. In the next step, extreme outliers were excluded from the analysis.

Lastly, all descriptive analyses were performed using Microsoft Excel. The results from the study include all selected European countries for each separate analysis. In addition, particular countries were selected as illustrative examples for each analysis.

### 2.4 Statistical analysis

It is hypothesized that countries with high market shares of Humira^®^ experience less pressure from price competition after biosimilar entry. The percentage change in price per DDD of Humira^®^ in countries with low market share of Humira^®^ was compared with that in countries with high market share of Humira^®^ using a parametric *t*-test for independent samples (also called two-samples *t*-test). Change in price per DDD in 2020 was calculated with reference to the year before biosimilar entry. In addition, market share of Humira^®^ 1 year before biosimilar entry was calculated with reference to the infliximab and etanercept markets combined. The differentiation between low and high market share of Humira^®^ was based on the median market share value of 38.0% (i.e., 50th percentile) in all countries. Countries with a market share equal to or higher than the median value were categorized in group 1 while countries with a lower market share were assigned to group 0. Market share of Humira^®^ was chosen as the independent variable and the change in Price per DDD of Humira^®^ as the dependent variable. The variables were first tested for normality using the Shapiro-Wilk test. The statistical analysis was performed using SPSS version 29 software. Selection of countries was based on the availability of Humira^®^ and adalimumab biosimilar(s) in 2020. In total 23 countries were selected for the analysis.

## 3 Results

### 3.1 First-mover advantage

**FIGURE 2:**
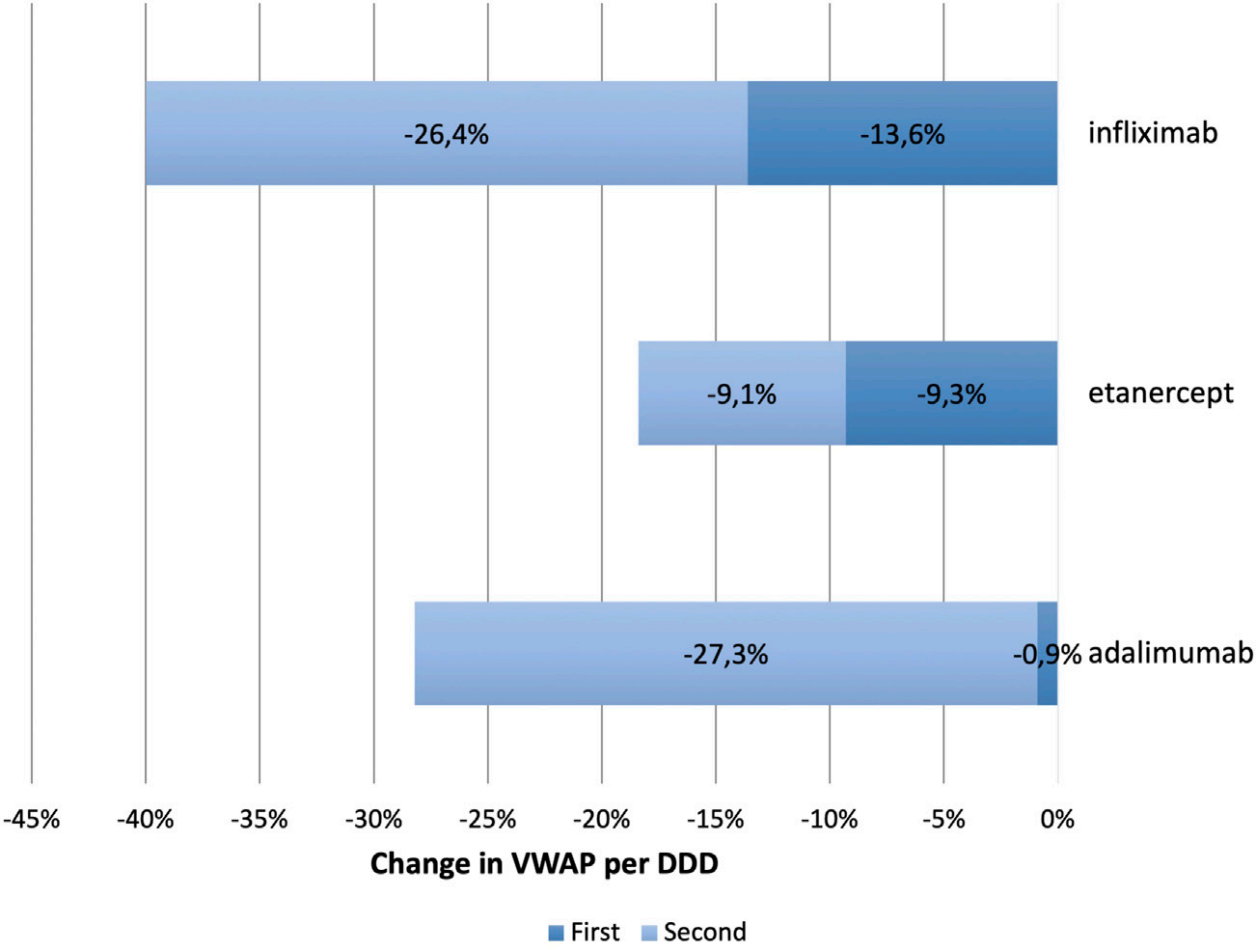
Average change in VWAP per DDD upon market entry of first and second biosimilar entrants for infliximab, etanercept, and adalimumab.

Figure 2 presents the average change in VWAP per DDD of infliximab, etanercept, and adalimumab at subsequent events of biosimilar entry. Further details regarding an overview of the percentage change in the VWAP per DDD and the number of biosimilar entrants at subsequent events of market entry across the selected European countries are included in Supplementary Table S1. Inclusion criteria were based on the occurrence of at least two or more events with biosimilar entry, and the availability of biosimilars for at least one of the three originator products Remicade^®^, Enbrel^®^, and Humira^®^. The number of included European countries for infliximab, etanercept, and adalimumab were 19, 9 and 15, respectively.

The number of biosimilars in the year during which the first biosimilar(s) entered the market was on average, 1.7 for infliximab, 1.1 for etanercept, and 2.3 for adalimumab. At the second event of biosimilar entry, this average remained the same for adalimumab (1.9) and etanercept (1.0) but decreased for infliximab (1.1).

With the first biosimilars arrival, the largest evolution in the VWAP per DDD was obtained for infliximab by an average of 13.6% ranging from an increase by 3.1% in Finland to a decrease by 33.6% in Lithuania. The second set of biosimilar entrants reduced the VWAP per DDD of infliximab on average by 26.4% ranging from 4.3% in Italy to 55.0% in Slovakia.

With the entry of the first biosimilar the VWAP per DDD of etanercept decreased the most by 9.3% on average, ranging from 1.8% in Germany to 13.7% in Poland. By the second event of biosimilar entry, a similar evolution in the VWAP per DDD as the first event was obtained, represented by an average of 9.1% ranging from an increase by 1.5% in Spain to a decrease by 24.0% in Switzerland.

Lastly with adalimumab, the first biosimilars showed a marginal effect on reducing the VWAP per DDD, more specifically with an average decrease by 0.9% ranging from an increase by 4.4% in Spain to a decrease by 9.7% in Slovenia. By the second event of biosimilar entry, the VWAP per DDD decreased the most for adalimumab by 27.3% on average ranging from 3.4% in Romania to 53.7% in Austria.

**FIGURE 3:**
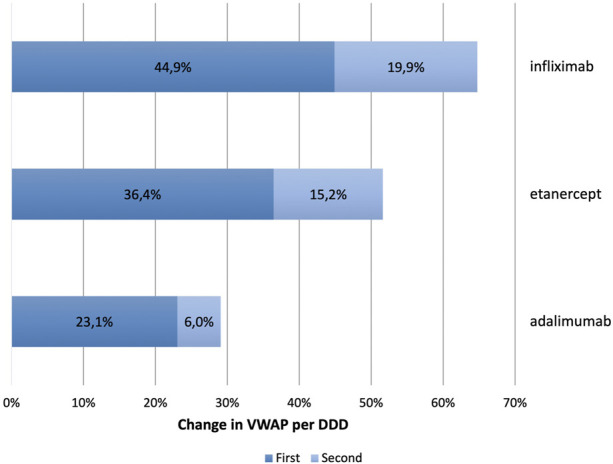
Average market share of first and second biosimilar entrants in 2020 for infliximab, etanercept, and adalimumab.

Market share in 2020 captured by the first and second set of biosimilars which entered the market at the first event of market entry, and the second event of market entry, respectively, differed among the three molecules, as shown in Figure 3. Market share of the first biosimilar entrants of infliximab was on average 44.9% ranging from 0.7% in Finland to 85.9% in Lithuania. For the second infliximab biosimilars, market share was on average 19.9% ranging from 0.2% in Norway to 83.1% in Finland. Across the selected countries, only in Slovenia, Finland, and Portugal, the second set of infliximab biosimilar captured a higher market share than the first ones. Within the class of etanercept, one biosimilar entered the market at each event, except in the United Kingdom where two etanercept biosimilars joined the market within the first event of entry. Across the selected European countries, average market share of the first biosimilar of etanercept was 36.4%, ranging from 13.6% in Austria to 73.9% in the United Kingdom. Market share of the second biosimilar of etanercept was on average 15.2% ranging from 0.3% in Sweden to 56.8% in Poland. In all countries, except for Poland, the first etanercept biosimilar captured the largest biosimilar market share. Finally, within the class of adalimumab, market share of the first biosimilars entering the market was on average 23.1% ranging from 4.3% in Austria to 76.3% in United Kingdom. The second biosimilar entrants captured a market share of 6.0% on average across the selected countries ranging from 0% in United Kingdom, Romania, and Ireland, to 16.9% in Slovenia. Across the selected countries, the second biosimilars of adalimumab achieved a higher market share only in Croatia, Slovenia, Austria, and Belgium.

### 3.2 Pricing strategies of originator companies

**FIGURE 4:**
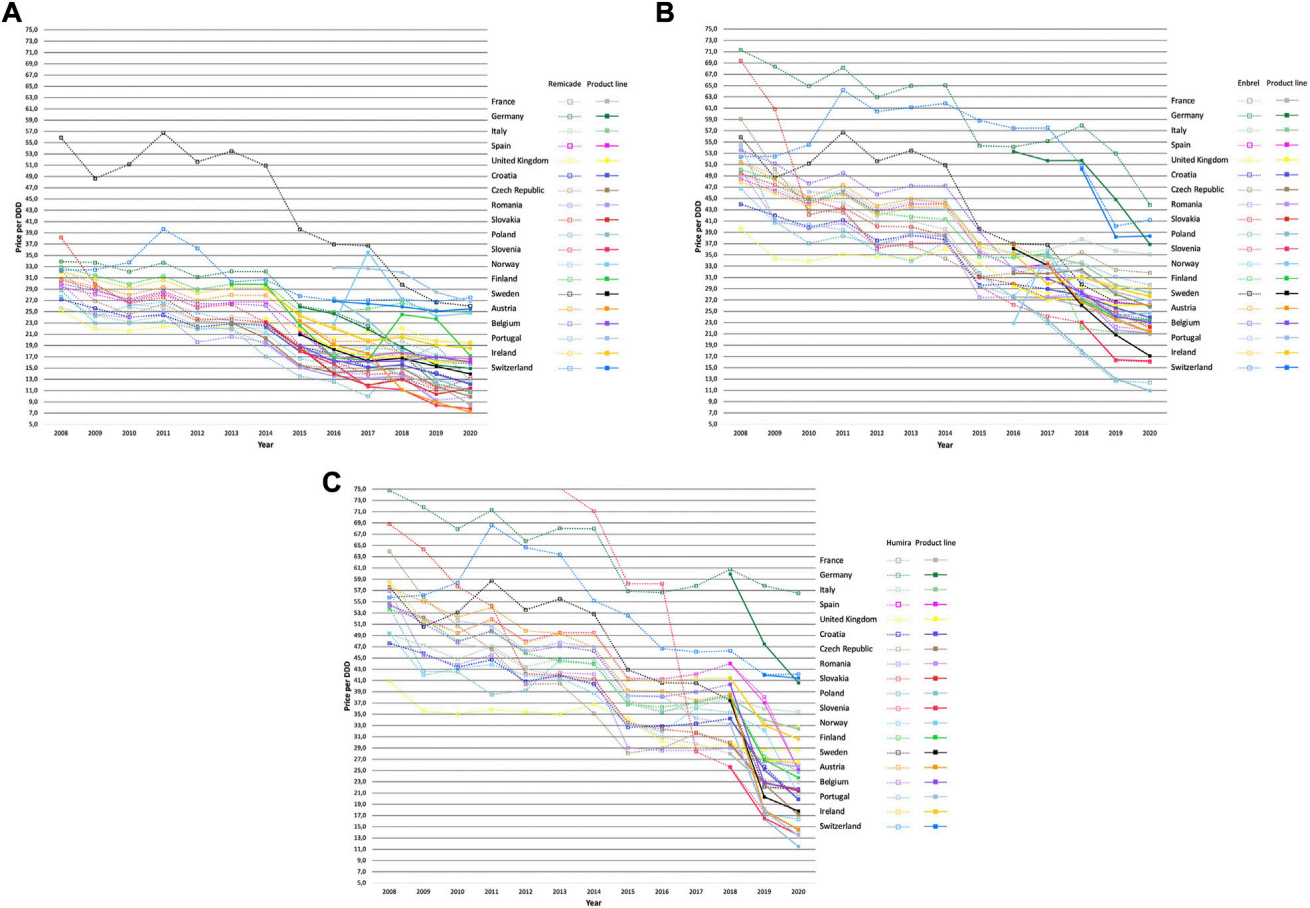
The evolution in price per DDD of the originator and VWAP per DDD of the product line (originator and its biosimilars) of infliximab **(A)**, etanercept **(B)** and adalimumab **(C)**.

Figure 4 presents an overview of the evolution of the price per DDD of the originator and the VWAP per DDD of the product line of infliximab, etanercept, and adalimumab in the selected European countries during the study period. Selection of countries was based on the availability of the originator as well as its biosimilars for all three molecules during the study period. Countries in which the originator biologic or any biosimilar exited the market for a certain period or indefinitely were excluded from the analysis. We selected for infliximab, etanercept, and adalimumab, 22, 20, and 24 European countries, respectively.

A prevailing pricing strategy can be observed as indicated by sharp price reductions from the originator adalimumab (Humira^®^) as a response to biosimilars entering the market (Figure 4C) in almost all selected countries. Compared to the latter, following the biosimilar entry, large list price reductions were to a great extent less noticeable with originator infliximab (Remicade^®^) (Figure 4A) and etanercept (Enbrel^®^) (Figure 4B). In addition, originator infliximab (Remicade^®^) showed relatively constant prices during pre- and post-launch of biosimilars as well which varied to a lesser degree within Europe in contrast to the other two TNF-alpha inhibitors. Originator etanercept (Enbrel^®^) and adalimumab (Humira^®^) also showed a similar trend in price evolution where the price per DDD of one biologic is consistently followed by the other throughout most of the study period in most of the European countries although the price per DDD of Enbrel^®^ decreased to a lesser extent compared to its competitor brand Humira^®^ following its loss of exclusivity.

**FIGURE 5:**
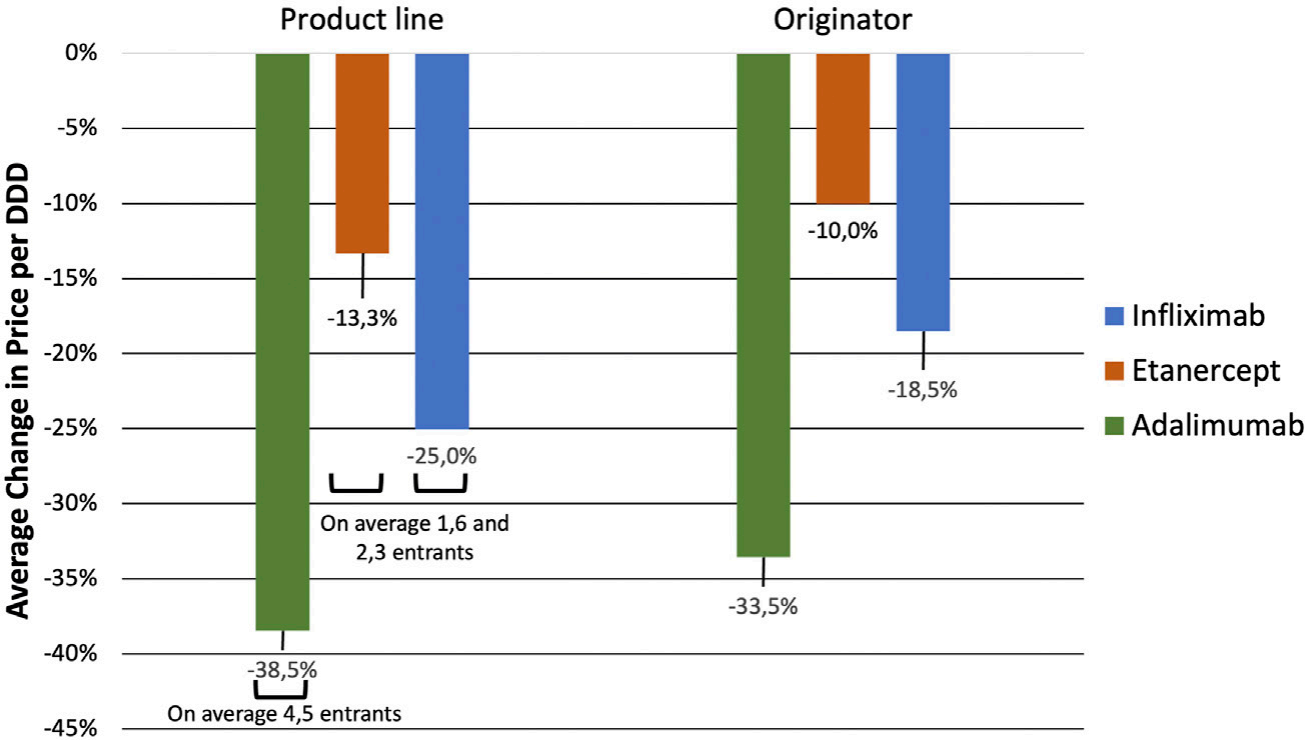
Average change in price per DDD of originator and the product line (originator and its biosimilars) 2 years since biosimilar entry versus year before entry for infliximab, etanercept, and adalimumab. The average number of available biosimilars of each molecule 2 years since biosimilar launch is presented as well.

In Figure 5, the average change in price per DDD of the originator and the VWAP per DDD (2 years since biosimilar entry versus year of biosimilar entry) and the average number of biosimilar entrants of each molecule in the selected countries are shown. The entrance of 2.3, 1.6, and 4.5 biosimilars on average was associated with a decrease, albeit to varying extent, in the average price of the originator biologic and the product lines of infliximab, etanercept, and adalimumab, respectively. Price per DDD of originator infliximab (Remicade^®^) decreased by 18.5% on average, ranging from −39.3% in Slovakia to a slight increase by 1.3% in Italy. Price per DDD of the originator etanercept (Enbrel^®^), decreased by 10.0% on average ranging from −36.0% in Poland to an increase by 11.7% in Czech Republic. Lastly, consequent to losing its market exclusivity, the price per DDD of originator adalimumab (Humira^®^) reduced on average the most among the three molecules, by 33.5%, ranging from −62.4% in Austria to −4.1% in United Kingdom. Furthermore, the VWAP per DDD of the product lines of infliximab, etanercept, and adalimumab, decreased by 25.0%, 13.3% and, 38.5%, respectively.

**FIGURE 6:**
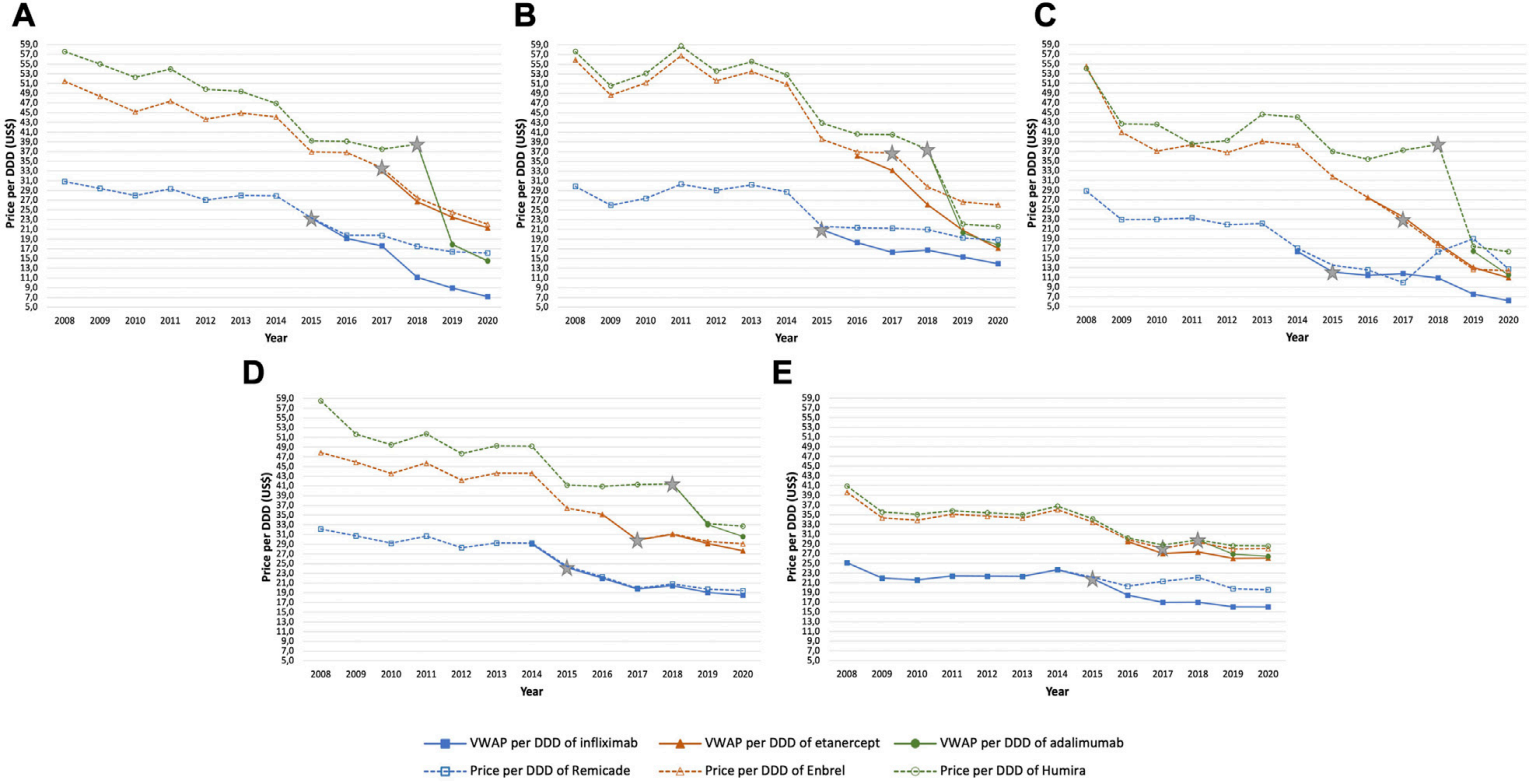
Evolution of the price per DDD of the originator biologic and VWAP per DDD for infliximab, etanercept and adalimumab in the following countries: Austria **(A)**, Sweden **(B)**, Poland **(C)**, Ireland **(D)**, and United Kingdom **(E)**. The market entry point of the first biosimilars is indicated with a star.

**FIGURE 7:**
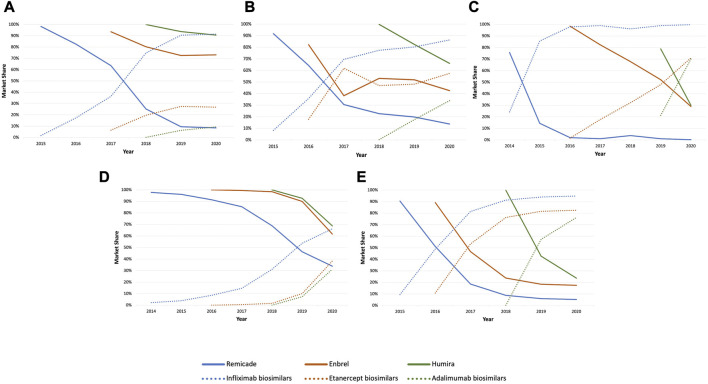
Market shares of originator and biosimilar infliximab, etanercept, and adalimumab, after biosimilar entry in the following countries: Austria **(A)**, Sweden **(B)**, Poland **(C)**, Ireland **(D)**, and United Kingdom **(E)**.

Figure 6 shows examples of trends in price per DDD of originator biologic and the product line following the arrival of biosimilars in selected countries. Alongside varying pricing responses from the originator biologics among the three molecules, variations in market shares, as shown in Figure 7 **,** were also observed indicating different rates at which originators lost their share of the market following the arrival of biosimilars.

In Austria (Figure 6A), price per DDD of originator adalimumab (Humira^®^) decreased the most among the three molecules after biosimilar entry. Market share of Humira^®^ (Figure 7A) dropped by only 10% since the introduction of biosimilars resulting in the originator retaining approximately 90% of the adalimumab market. This is also displayed in Figure 6A where the curves of the originator and the product line nearly overlap suggesting that price reductions from the originator (Humira^®^) led to it retaining a fixed share of the adalimumab market. Similar patterns of pricing strategies can be seen in, Belgium, Portugal, Luxembourg, Finland, and Croatia, among others. On the other hand, the case with originator infliximab (Remicade^®^) and etanercept (Enbrel^®^) illustrate a different trend in pricing strategies in Austria. Price per DDD of Remicade^®^ decreased modestly after biosimilar entry which resulted in the originator losing approximately one-fifth of the market. This is again, demonstrated in Figure 6A **,** where the curves of the originator and the product line did not overlap, indicating a market shift to biosimilars. A slight increase in the price per DDD of the originator infliximab the next year, in 2017, resulted in a steeper decrease in its market share compared to previous years. Price response from originator etanercept (Enbrel^®^) was akin to originator infliximab after biosimilar entry. Contrary to Remicade^®^, the curves of the VWAP per DDD of etanercept and Enbrel^®^ nearly overlap suggesting that the originator retained most of the market. This is indicated by a less steep decrease in market share of Enbrel^®^, as shown in Figure 7A.

In Sweden (Figure 6B), significant price reductions from Humira^®^ like in Austria were observed. However, the originator lost its market share at a higher speed. As for Enbrel^®^, no observable decreases but rather an increase in price per DDD was found upon the entry of biosimilars, leading to the originator etanercept losing two-third of its share of the market (Figure 7B). However, a volume shift from its biosimilar counterparts back to Enbrel^®^, was observed following noticeable price reductions after. Lastly, the evolution in the price per DDD of Remicade^®^ in Sweden showed a general trend of stabilization for 3 years since biosimilar entry, leading to a decrease of nearly 80% of the originators’ share of the market. In Poland (Figure 6C), similar to Sweden, a significant decrease in price per DDD of Humira^®^ did not result in the originator retaining a considerable share of the market (Figure 7C).

In Ireland, price per DDD of Humira^®^ decreased the most upon biosimilar entry among the three molecules leading to the originator retaining almost all its share of the market as indicated by an overlap in the curves of the originator and the product line (Figure 6D). However, a stabilization in the price per DDD of the originator resulted into a steep increase in market share of its biosimilar counterparts (Figure 7D). Additionally, market share of adalimumab and etanercept biosimilars evolved in a synchronized fashion.

In the United Kingdom, no profound changes in price per DDD of any originator biologic (Figure 6E **)** following biosimilar entry was observed. Interestingly, market share of biosimilars (Figure 7E) increased in a considerable high rate following market entry. A similar trend was also observed in Italy.

Lastly, we analyzed the impact of biosimilar competition on change in list price per DDD of Humira^®^ in countries with low market share of Humira^®^ (year before biosimilar entry) versus countries with high market share using an independent samples *t*-test. In total 11 countries were included in group 0 (i.e., low market share) and 12 countries in group 1 (i.e., high market share), with 48% and 52% of the sample size, respectively. Since biosimilar entry, price per DDD of Humira^®^ decreased on average by 35.3% in countries with low market share, and by 28.4% in countries with high market share of Humira^®^ across the selected countries. The results of the independent samples *t*-test showed a non-significant difference (*t*-test: 0.723, *p* > 0.05) in change in price per DDD of Humira^®^ between countries with low and high market share before entry of biosimilars, indicating that the means of the two groups are equal.

### 3.3 Utilization trends

Table 1 shows an overview of the overall percentage change in volume, thus utilization since biosimilar entry, the average annual change in volume before (i.e., pre-launch) and after (i.e., post-launch) introducing biosimilars to the market, for the three molecules infliximab, etanercept and adalimumab. In total 25 European countries were selected based on the availability of biosimilars during the study period and out of those 22, 19, and 22 countries were selected for infliximab, etanercept, and adalimumab, respectively. The results demonstrate that since biosimilar entry the average utilization (expressed as DDDs per 1,000 inhabitants per day in each country) of the three anti-TNF alpha inhibitors increased significantly in most, but not all countries.

**TABLE 1:** Percentage change in volume, average annual change in volume pre-launch, and post-launch, of infliximab, etanercept and adalimumab in European countries.

	Change in volume (2019 versus year before biosimilar entry) (%)			Average annual change pre-launch (%)			Average annual change post-launch (%)		
	Infliximab	Etanercept	Adalimumab	Infliximab	Etanercept	Adalimumab	Infliximab	Etanercept	Adalimumab
France	62.0%	−4.8%	23.5%	11.3%	4.0%	13.2%	10.2%	−1.2%	11.1%
Germany	29.9%	33.6%	25.2%	13.7%	5.3%	11.9%	5.4%	7.6%	12.1%
Italy	16.4%	0.0%	14.5%	1.9%	1.0%	9.4%	3.1%	0.1%	7.0%
Spain	37.2%	10.7%	29.7%	6.4%	6.3%	9.1%	6.6%	2.6%	14.0%
United Kingdom	67.9%	14.6%	11.9%	8.9%	3.8%	13.6%	10.9%	3.8%	5.8%
Austria	110.9%	26.2%	30.3%	12.9%	139.8%	121.6%	19.6%	8.4%	14.3%
Belgium	66.5%	5.8%	17.6%	5.7%	5.2%	6.0%	10.8%	1.9%	8.0%
Ireland	160.6%	−6.0%	15.6%	12.8%	5.2%	12.3%	17.3%	−1.4%	7.5%
Luxembourg		−26.5%	14.9%		2.8%	6.1%		−9.7%	14.9%
Switzerland	29.7%	−1.6%	5.7%	9.1%	4.4%	6.9%	6.7%	−0.8%	5.7%
Portugal	146.4%	11.0%	26.0%	4.0%	8.3%	16.3%	13.8%	2.7%	12.2%
Croatia	186.7%	39.0%	55.2%	19.7%	18.4%	22.7%	19.4%	11.9%	26.3%
Czech Republic	85.0%	39.9%	26.1%	−6.9%	4.0%	22.6%	9.9%	8.8%	12.3%
Latvia	115.0%		16.3%	15.7%		136.6%	19.4%		16.3%
Bosnia	170.6%			31.0%			29.9%		
Bulgaria			19.7%			55.9%			19.7%
Hungary			13.9%			11.8%			13.9%
Romania	−14.1%	14.1%	1.7%	19.8%	12.1%	24.5%	−2.2%	5.4%	1.7%
Serbia	161.9%			30.2%			28.6%		
Slovakia	−17.2%		−1.7%	49.9%		26.0%	1.9%		−1.7%
Slovenia	72.8%	6.3%	20.5%	21.1%	5.7%	28.2%	11.6%	3.3%	9.8%
Poland	246.0%	23.4%	21.3%	6.0%	13.0%	28.0%	25.4%	5.6%	21.0%
Norway	167.2%	16.8%		5.9%	0.8%		15.5%	4.3%	
Finland	11.4%	23.1%	54.1%	12.9%	2.0%	5.0%	14.2%	12.4%	24.3%
Sweden	43.4%	52.2%	49.9%	6.7%	2.0%	9.1%	7.5%	11.2%	23.7%
AVERAGE	**88.9%**	**14.6%**	**22.4%**	**13.6%**	**12.8%**	**27.1%**	**13.0%**	**4.0%**	**12.7%**

The bold values in Table 1 are the average values of each column.

The average change in volume since biosimilar entry for the selected countries was highest for infliximab, followed by adalimumab and etanercept. After detection and removal of extreme outliers from the analysis, this order remained unchanged (results are available from corresponding author upon request). First, utilization of infliximab increased by 88.9% on average since biosimilar entry ranging from an increase by a factor of approximately 3.5 (246%) in Poland to a decrease by 17% in Slovakia. Utilization of etanercept showed a 14.6% increase on average ranging from an increase by a factor of 1.5 (52%) in Sweden to a decrease by 27% in Luxembourg. Finally, utilization of adalimumab increased 22.4% by average ranging from an increase by a factor of 1.5 (55%) in Croatia to decrease of 1.7% in Slovakia.

Overall, since the entry of biosimilars, increase in utilization was significantly higher in Central and Eastern European markets compared to Western European markets. In countries like Croatia, Poland, and Czech Republic, significant increase in utilization of infliximab, etanercept, and adalimumab, was detected.

Some European countries require further attention. For instance, after biosimilar entry, the average annual change in volume pre-launch versus post-launch remained unchanged in most EU4 countries and United Kingdom. Examples of the latter include France (11.3% versus 10.2%), Italy (1.0% versus 0.1%), and Germany (11.9% vs. 12.1%), with infliximab, etanercept, and, adalimumab, respectively. On the other hand, a higher average annual change in volume was observed following biosimilar arrival (i.e., post-launch) of the three molecules in all included Scandinavian countries (Sweden, Finland, and Norway), compared to the period prior biosimilar entry. Increase in utilization one molecule coupled with a decrease or no change in utilization of the other two molecules was also observed after the entry of biosimilars. Only within the class of infliximab the average annual change in volume increased following the introduction of biosimilars in Poland (6.0%–25%), Latvia (15.7%–19.4%), Ireland (12.8%–17.3%), United Kingdom (8.9%–10.9%) Austria (12.9%–19.6%), Italy (1.9%–3.1%), and Portugal (4.0%–13.8%). An increase in the average annual change in volume only within the class of etanercept among the three molecules was observed in Germany (5.3%–7.6%). In Croatia, the average annual change in volume decreased with infliximab and etanercept but increased (22.7%–26.3%) with adalimumab achieving the highest average annual change in volume of adalimumab post-launch out of all included countries. Finally, a decreased average annual change in volume of each molecule was observed in Romania, Slovakia, Slovenia, Serbia, Bosnia, Bulgaria, and Switzerland. Particularly, in Romania the average annual percentage change in volume of infliximab post-launch of biosimilars was negative.

## 4 Discussion

In this study, trends in originator and biosimilar prices, market shares and utilization were investigated for infliximab, etanercept, and adalimumab from 2008 to 2020 in European countries. We also explored the existence of a biosimilar first-mover advantage and whether first biosimilar entrants spur the largest price decrease in the product line (originator and its biosimilars) while benefitting from a market share advantage vis-à-vis second or late entrants. We found that within all three TNF-alpha product lines, the first biosimilar(s) captured on average a long-standing higher market share than the second biosimilar entrant(s) but this did not result in the largest decrease in the VWAP per DDD. Another finding was price reductions of different magnitudes among the three molecules suggesting influence of varying competitive pricing strategies exhibited by the originator companies. We also observed dominant patterns of large reductions in the price of originator adalimumab (Humira^®^) as a response to biosimilar entry in most countries. Next to price reductions, this paper has shown that increase in overall utilization did occur following biosimilar entry. However, the strength of the relationship between price reductions and increase in access varied significantly among the three molecules and across European markets, as we will discuss below.

### 4.1 First-mover advantage

Following patent expiry and loss of market exclusivities of the originator brand, entrance of lower-priced biosimilars stimulates competition in the off-patent biologics market (Mulcahy et al., 2018). Being the first biosimilar manufacturer is an important determinant for the market share. Our findings confirm our hypothesis that he first biosimilar manufacturer(s) can seize a window of opportunity to benefit from a substantially market share advantage against second or late biosimilar entrants (Endrenyi et al., 2017). These findings seem to be consistent with other research in selected European countries which reported specifically for biosimilars within the class of TNF-alpha inhibitors that the first biosimilars entering the market could retain more than 70% in some biosimilar markets (Troein et al., 2017). Furthermore, with increasing number of biosimilar competitors entering the market as first or second entrants, the cumulative market share of these biosimilars might even exceed originator’s market share, as we’ve seen in the United Kingdom for instance where the first biosimilars of etanercept and adalimumab captured more than 70 percent of the market.

In addition, the present study has demonstrated that the first biosimilar(s) entering the market can benefit from smaller price reductions for a certain amount of time before second or late entrants join the market driving down prices further, as seen with infliximab and adalimumab. Therefore, overall findings from present study suggest that increasing number of competitors enhances competition and the effect on price reductions. However, experience from Europe has shown a weak correlation between price reductions of the total market and the number of biosimilars referring to the existence of barriers to effective competition and adding that cost-savings from biosimilar competition can be achieved without the presence of multiple biosimilars (Troein et al., 2017; OECD, 2018). However, in the present study we show that entry of (multiple) biosimilars following the second event of entry usually resulted in a greater effect at reducing the VWAP per DDD for infliximab and adalimumab, underpinning that the market entry of more biosimilars is essential to promote price competition, and hence cost-savings. Nonetheless, the heterogeneity in the size of price reductions and the market captured by the first and second biosimilar entrants observed in present study limit generalizability of the results. Therefore, it is important to take this heterogeneity into consideration when interpreting EU averages which mask considerable variations in list price changes among European countries. It is significant to note that within the class of etanercept, a marginal difference (−9.3% and −9.1%) was found in average price reductions of the VWAP per DDD in both events of biosimilar entry with the same average number of biosimilars (1 and 1) entering the market at each event. A possible explanation might be the low number of available etanercept biosimilars.

### 4.2 Pricing strategies from originator companies

Another determinant of biosimilar uptake is how list prices of the originator brand might behave as a response to biosimilar entry. Recent evidence from Europe suggests that strategies from originator companies to protect their market share may have shifted more from traditional strategies such as product modifications, second generation products, and supporting devices, towards price competition (Troein et al., 2019). In the present study, we have identified archetypal pricing strategies that originator companies may have used which serve as pricing models that can be adapted throughout the price evolution of a pharmaceutical. On one hand, the originator company may decide not to react distinctively by not competing excessively on the list price, as seen with the originator infliximab (Remicade^®^) and etanercept (Enbrel^®^). The latter pattern of pricing behavior has resulted in a volume shift from the originator to its biosimilars in most countries. On the other hand, the originator company may offer significant list price reductions to avoid market penetration of its biosimilar competitors leaving an unattractive market to present and future biosimilar manufacturers. The dominant pattern of aggressive price cuts observed with Humira^®^ in several countries is one example of the latter. This finding is consistent with an online newspaper article in which large discounts as high as 80% tied to volume were reportedly offered by AbbVie to hospitals in the Netherlands on condition that all patients will be treated with the originator biologic (Hordijk, 2019). Various practices adapted by originator companies considered to be anti-competitive resulted in numerous examples of thorough investigation by European competition authorities (CMA, 2019; Hordijk, 2019; Autoriteit Consument and Markt, 2022). It has been suggested that the slow uptake of the first adalimumab biosimilar entrants compared to early launches as seen with infliximab, etanercept, rituximab, and trastuzumab, might be a result from large discounts and long-lasting contracts offered by AbbVie to protect its share of the market (Arias, 2019). These results support our finding that in most countries the originator adalimumab (Humira^®^) succeeded in retaining its market share to a certain extent. Considering that price reductions observed in present study are based on ex-manufactory level, it is expected that due to confidential discounts net prices might decrease even further. This important finding supports the idea how aggressive pricing strategies may negatively impact biosimilar entry eventually leading to biosimilar manufacturers opting out of the market. Subsequent market dominance of the originator brand has the potential to reduce healthcare payers’ bargaining power and cause issues like supply constraints as well. Additionally, conditional discounts also limit switching patients to biosimilars since healthcare payers face losing all cost-savings bound to the use of the originator brand.

### 4.3 Shadow pricing

Of note in this study is how intravenous (infliximab) and subcutaneous (etanercept and adalimumab) TNF-alpha inhibitors exhibited different trends in price evolution. Originator etanercept (Enbrel^®^) and adalimumab (Humira^®^) showed similar trends in price evolution: the price per DDD of both products evolved in the same fashion. This trend may reflect “shadow pricing”, where instead of price reductions to gain market share from one another both competing manufacturers mirror each other’s price changes consistently keeping prices high without a well-grounded justification (Silverman, 2017). This has been seen in the US where a price increase of 9.7% of Humira^®^ was mirrored by an identical price increase by its competitor brand Enbrel^®^ (Drug Pricing Investigation, 2020). It has been argued as well that the practice of shadow pricing is associated with payers’ and customers’ willingness to pay (Rathore and Shereef, 2019). Since both products are administered subcutaneously and are each other’s primary brand competitors in rheumatoid arthritis, future entry of adalimumab biosimilars in turn might influence any (decreasing) change in price of Enbrel^®^ as well.

### 4.4 Supply-side and demand-side policies

Next to competition strategies of originator brands, market penetration of biosimilars might be influenced by government intervention in terms of supply-side (e.g., mandatory price cuts) and demand-side policy measures (Rémuzat et al., 2017b; Moorkens et al., 2017). Pricing systems for biosimilars in European countries can be identified by government regulated prices, free pricing, or a combination of both. However, it is important to bear in mind that the observational character of the current study does not allow to take into account the extent that price reductions following biosimilar entry may be influenced by specific pricing systems. When relying solely on supply-side policies, it becomes clear that for healthcare payers the resulting competition from market entry of biosimilars is mainly price-driven by pursuing cost-savings from lower priced alternatives and additional (confidential) discounts. However, although price regulation such as mandatory price decreases result in cost-savings, biosimilar competition may be negatively affected in long-term. The lower market uptake of adalimumab biosimilars in countries like Austria, Ireland, and Belgium in present study, could be explained partially by the sharp price reductions from Humira^®^ as a response to biosimilar entry coupled with cost-containment measures with mandatory price cuts. Although these practices generate savings in short-term, a persistent low biosimilar uptake may in turn lead to fewer biosimilar developed in the future.

Next to these supply-side measures, payers can launch additional incentives to promote uptake of biosimilars in Europe. Examples of demand-side policies have been observed in countries such as France, Germany, Ireland, and Belgium (Reilly and Schneider, 2020). These incentives aim to encourage physicians to prescribe the least expensive biologic and in turn increase the price competition among manufacturers.

The best-value biological program was introduced in 2019 in Ireland with the prospect to increase the uptake of adalimumab biosimilars Imraldi^®^ and Amgevita^®^ and etanercept biosimilar Benepali^®^ by issuing financial prescribing incentives (e.g., gain-share of €500 per patient) when initiating treatment with naïve patients or switching. Interestingly, our results indicate a steeper increase in the uptake of etanercept and adalimumab biosimilars after 2019 which mirror those of a previous study examining the impact of the best‐value biological medicine initiative on the uptake of biosimilars for adalimumab and etanercept. This Irish program resulted in cost-savings of approximately 22.7 million 1 year following the launch (June 2019) with the proposed best-value biologics covering half of the market (Duggan et al., 2021).

Similarly in Belgium, financial incentives to stimulate biosimilar uptake in ambulatory care were introduced to prescribing physicians in January 2019 (Vandenplas et al., 2021). If a certain quota (5%, 10% or 20%) of biosimilar prescriptions of adalimumab and etanercept combined were reached, physicians could receive a financial reward. Published results (June 2019) of this initiative indicated hardly an effect as shown by market reach of 7.1% by adalimumab biosimilars and 16.5% by etanercept biosimilars since market entry in 2018 (adalimumab) and 2016 (etanercept) (Medaxes, 2019). These results match those observed in our study suggesting that the success of this Belgian initiative on increasing market share of these biosimilars was rather minimal (Vandenplas et al., 2022).

The practice of pharmacy-level substitution of generic medicines is widely applied in the EU Member States, except for Austria and the United Kingdom, and has shown an immense positive impact on their increased uptake (Vogler et al., 2021). Contrarily in the EU, biologic substitution practices or interchangeability designation falls within the responsibility of the Member States, and thus are not regulated on an EU level. Pharmacy-level substitution of biologic medicines, including biosimilars, is therefore not a common practice in almost all Member States in the EU. Recently, EMA has published for the first tiem a statement on interchangeability announcing that a biosimilar medicine approved by EMA is interchangeable with its reference product or an equivalent biosimilar medicine (EMA, 2022). This change in regulatory approach by EMA is considered to open a window of opportunity for national medicines agencies to implement changes in their legislations regarding automatic substitution of biologic medicines which can in turn positively influence uptake of biosimilar medicines.

### 4.5 Patient access

An important benefit from biosimilar competition is increased access to biologic treatments which will result into improved health outcomes. Especially for the region of CEE, where access restrictions to biologic treatments are frequently reported, increased utilization is a key benefit from biosimilar competition (Inotai et al., 2018). In the present study, the effect of biosimilar entry on usage of biologics was significantly higher in CEE markets compared to Western European markets. This substantial increase in the region of CEE can be attributed to historically low usage of biologics before biosimilar entry (IQVIA, 2021). However, despite a significant increase in utilization, a historical low usage of biologic treatments implicates a limited opportunity for biosimilars in generating sufficient cost-savings (Inotai and Kaló, 2019; Troein et al., 2020). Therefore, it has been argued that adopting more restrictive eligibility criteria, delaying reimbursement or shorter duration of treatment and the slow economic growth are key factors that limit access to protected biologic treatments in the CEE region resulting in low usage of biologic treatments (Orlewska et al., 2011; Inotai et al., 2018; Inotai and Kaló, 2019). However, this widens the inequity in treatment access and health gap between CEE and Western Europe. The introduction of multiple biosimilar competitors generates a positive impact on utilization of biologic treatments, but the present study confirmed that this does not necessarily translate into growth in treatment access for each product line (originator and its biosimilars). Our findings showed decrease in utilization of some or all molecules following biosimilar entry in countries like Croatia, and Romania despite observed large reductions in the VWAP per DDD. These results agree with the findings of other European studies in which decrease in utilization of some biologics have been reported after market entry of biosimilars which might be attributed to increase in utilization of another molecule (IQVIA, 2020; Barszczewska and Piechota, 2021; Tachkov et al., 2021). For instance, a study in Bulgaria reported decrease in utilization of infliximab which was attributed to constant price reductions and entry of new biosimilars within the group of TNF-alpha inhibitors such as adalimumab which is a commonly prescribed TNF-alpha inhibitor (Tachkov et al., 2021).

All Scandinavian countries (Sweden, Finland, and Norway) included in this study showed increase in utilization of each molecule as indicated by a higher average annual change in volume post-launch of biosimilars in contrast to pre-launch. The present findings seem to be consistent with other research which found increase in growth rate of utilization of TNF-alpha inhibitors in Scandinavian markets after biosimilar entry even though high usage of biologics already existed before the arrival of biosimilars (Troein et al., 2019). Scandinavian countries have been leading the way in Europe in terms of switching and quick and early adoption of biosimilars, hence, increased patient access to biologic therapies and the cost savings achieved (Madsen, 2017). One of the driving forces for the strong uptake of biosimilars in this region is the positive view of all stakeholders on biosimilars, clear dissemination of information, and the strong engagement of prescribing physicians during the procurement process and throughout tendering discussions (Azuz et al., 2021; Barbier et al., 2021).

To our knowledge, this is the first study investigating the first-mover advantage of biosimilars across multiple European markets. In addition, the present study succeeded to enhance our understanding how pricing strategies from originator companies, specifically aggressive discounting, might affect the competitive environment of off-patent biologics and biosimilars market.

### 4.6 Limitations

This study has also several limitations. First, data used for the analysis do not represent net prices and, hence, do not consider (confidential) discounts for the retail and hospital sector. Therefore, the authors could not make accurate conclusions regarding market dynamics based on absolute net prices. Secondly, since volume data were described in the IQVIA database as the number of packages (units) sold in each country; issues such as parallel exporting could not be identified or adjusted for. In addition, data provided by IQVIA were classified on a yearly basis meaning that the first biosimilar entrant could not be determined for each molecule in almost all European countries. For instance, Remsima^®^ or Inflectra^®^, known as the first approved infliximab biosimilar CT-P13, were independently marketed by Celltrion and Hospira (now Pfizer), respectively, and joined the market at the same time in most but not all European countries as seen in present study. Furthermore, no data were available on indications or diagnosis. The TNF-alpha inhibitors markets do not represent a unified market which can function differently by indication, for example, rheumatoid arthritis where more treatments options are available and inflammatory bowel disease where severe consequences can occur with treatment failure. Therefore, indication information is needed to evaluate differences in market dynamics accurately. Lastly, considering different expiration dates of the market exclusivities of originator brands, another limitation occurred mainly in the case for adalimumab since the patent of the originator brand expired in October 2018 indicating that only a maximum of 2 years of data since biosimilar entry could be obtained to evaluate.

### 4.7 Future research

Further research should be done to extend our analysis with net prices from originator and biosimilar products to gather more precise insights into differences in market dynamics between molecules and countries. This might be especially of interest in countries where tender systems are frequently in place to procure biosimilars and biologic medicines. Also, further research might be of interest to explore any market shifts to innovative medicines within or outside the class of TNF-alpha inhibitors. Lastly, it might be interesting to gain additional insights about the first-mover advantage based on data containing explicit timepoints of market entry which enables to distinguish the first biosimilar entrant from the subsequent entrants rather than sales and volume data classified on a yearly basis.

## 5 Conclusion

In this study, a varying landscape of opportunities for biosimilars regarding patients access and sustainability for the drug budget can be seen. This study has shown that the extent of price competition varies between molecules and European countries and depends on the year following patent expiration during which biosimilars can enter the market. First, observed trends in market share and price (earnings) clearly indicate a biosimilar first-mover advantage. Second, competition can be seen between the three molecules with three pricing archetypes which determine the chance for successful entry of biosimilars in a certain market. Third, lower priced biosimilars offer in general the possibility of greater patient access indicating that more patients can be treated as well as initiating treatment in the earlier phase of their disease. Also in this matter, a large variation in patient access by molecule and country or healthcare system can be observed. We conclude that in Europe a large discrepancy is present in how countries are able to capture the benefits of biosimilars. Authorities should take this information into account to develop policy measures targeting different market dynamics to ensure cost-savings from biosimilar competition in long-term. Therefore, healthcare payers should aim to achieve a level-playing field that supports the market participation of multiple (originator and/or biosimilar) manufacturers to ensure a sound bargaining power–beyond price alone—in the presence of various alternative sources of biologic medicines.

## Data Availability

The data analyzed in this study is subject to the following licenses/restrictions: The dataset is restricted to use by the authors of this manuscript. It may be available upon reasonable request. Requests to access these datasets should be directed to elif.car@kuleuven.be.
