# Supplementary material for: Biosimilar competition in European markets of TNF-alpha inhibitors: a comparative analysis of pricing, market share and utilization trends
Source: Front Pharmacol. 2023 Apr 21;14:1151764. doi: 10.3389/fphar.2023.1151764 (PMC10160635; doi:10.3389/fphar.2023.1151764)
Supplement: Supplementary file 1 [file Table1.DOCX]

Supplementary Material

Biosimilar competition in European markets of TNF-alpha inhibitors: a comparative analysis of pricing, market share and utilization trends

Elif Car^1*^, Arnold Vulto^1,2^, Mark Van Houdenhoven^3^, Isabelle Huys,^1^ Steven Simoens^1^

*** Correspondence:** Corresponding Author: elif.car@kuleuven.be

**Supplementary Table 1:** Change in VWAP per DDD (current event versus previous event) and the number of biosimilar entrants on first and second event with biosimilar entry for infliximab, etanercept, and adalimumab.

**TABLE 1**. Change in VWAP per DDD (current event versus previous event) and the number of biosimilar entrants on first and second event with biosimilar entry for infliximab, etanercept, and adalimumab.

|  | **First event of market entry** | | | | | | **Second event of market entry** | | | | | |
| --- | --- | --- | --- | --- | --- | --- | --- | --- | --- | --- | --- | --- |
|  | **Change in the VWAP per DDD (%)** | | | **Number of biosimilar entrants** | | | **Change in the VWAP per DDD (%)** | | | **Number of biosimilar entrants** | | |
|  | **Infliximab** | **Etanercept** | **Adalimumab** | **Infliximab** | **Etanercept** | **Adalimumab** | **Infliximab** | **Etanercept** | **Adalimumab** | **Infliximab** | **Etanercept** | **Adalimumab** |
| **France** | -23,5 | -11,7 | -5,9 | 2 | 1 | 2 | -10,5 | -0,7 | -19,1 | 2 | 1 | 3 |
| **Germany** | -19,5 | -1,8 | 3,7 | 2 | 1 | 4 | -4,8 | -3,1 | -20,8 | 1 | 1 | 1 |
| **Italy** | -17,6 | -5,0 | 4,1 | 2 | 1 | 2 | -4,3 | -5,1 | -10,1 | 1 | 1 | 1 |
| **Spain** | -19,8 | -7,7 | 4,4 | 2 | 1 | 2 | -20,5 | 1,5 | -15,8 | 1 | 1 | 2 |
| **UK** | -7,8 | -11,9 | 3,5 | 2 | 2 | 3 | -15,5 | -8,4 | -9,8 | 1 | 1 | 1 |
| **Croatia** | -2,7 |  | 2,7 | 1 |  | 2 | -19,4 |  | -26,6 | 1 |  | 3 |
| **Czechia** | -1,2 |  | -6,2 | 1 |  | 1 | -12,1 |  | -23,9 | 1 |  | 3 |
| **Lithuania** | -33,6 |  |  | 1 |  |  | -45,2 |  |  | 1 |  |  |
| **Romania** | -4,9 |  | -8,4 | 2 |  | 3 | -53,6 |  | -3,4 | 1 |  | 1 |
| **Slovakia** | -2,3 |  |  | 2 |  |  | -55,0 |  |  | 1 |  |  |
| **Poland** | -26,1 | -13,7 |  | 2 | 1 |  | -33,3 | -14,3 |  | 1 | 1 |  |
| **Slovenia** | -22,1 |  | -9,7 | 1 |  | 2 | -35,0 |  | -35,2 | 1 |  | 2 |
| **Norway** | -6,2 |  |  | 1 |  |  | -15,8 |  |  | 1 |  |  |
| **Finland** | 3,1 |  | 2,8 | 2 |  | 2 | -42,5 |  | -29,4 | 1 |  | 1 |
| **Sweden** | -27,0 | -8,8 | -7,6 | 2 | 1 | 2 | -27,0 | -8,3 | -45,8 | 2 | 1 | 3 |
| **Austria** | -16,6 | -10,3 | 2,7 | 2 | 1 | 2 | -52,1 | -19,2 | -53,7 | 1 | 1 | 2 |
| **Belgium** | -27,2 |  | 3,5 | 2 |  | 2 | -10,7 |  | -43,5 | 1 |  | 3 |
| **Portugal** | -2,9 |  | -3,2 | 1 |  | 2 | -12,3 |  | -46,7 | 1 |  | 2 |
| **Ireland** | -0,6 |  | 0,4 | 2 |  | 3 | -31,9 |  | -26,2 | 1 |  | 1 |
| **Switzerland** |  | -12,7 |  |  | 1 |  |  | -24,0 |  |  | 1 |  |
| AVERAGE | -13,6 | -9,3 | -0,9 | 1,7 | 1,1 | 2,3 | -26,4 | -9,1 | -27,3 | 1,1 | 1,0 | 1,9 |
